# The role of vascular pathology in SMA: implications for pathogenesis and treatment

**DOI:** 10.3389/fncel.2026.1933459

**Published:** 2026-10-06

**Authors:** Simon H. Parson, Hazel Allardyce

**Affiliations:** 1 Institute of Education in Healthcare and Medical Sciences, School of Medicine, Medical Sciences and Nutrition, University of Aberdeen, Aberdeen, United Kingdom; 2 School of Infection and Immunity, College of Medical, Veterinary and Life Sciences, University of Glasgow, Glasgow, United Kingdom

**Keywords:** blood spinal cord barrier, blood vessel, endothelial dysfunction, hypoxia, motor neuron disease, neurodegeneration, spinal muscular atrophy, vascular biomarker

## Abstract

Spinal muscular atrophy (SMA) has become a paradigm for precision medicine following the development of transformative therapies targeting the disease-causing deficiency of survival motor neuron (SMN) protein. As more than 98% of SMA cases arise from mutations in the *SMN1* gene, restoration of SMN expression through nusinersen (Spinraza; Biogen, Cambridge, MA), risdiplam (Evrysdi; Roche, Basel, Switzerland) and onasemnogene abeparvovec (Zolgensma and Itvisma; AveXis, Novartis, Chicago, IL) has produced unprecedented clinical benefits and established SMA as the first treatable motor neuron disease. To date, therapeutic success has focused on rescuing motor neurons, the principal site of clinical pathology. However, growing evidence indicates that SMA is a multisystem disorder in which pathological changes extend beyond the motor unit. In particular, vascular pathology has emerged as a recurring feature across numerous affected tissues. Vascular abnormalities have been identified throughout the neuromuscular system, including the spinal cord and skeletal muscle, as well as in systemic organs such as the heart, kidney, gastrointestinal tract and spleen. Experimental studies suggest that endothelial dysfunction is a direct consequence of SMN deficiency, impairing angiogenesis, vascular maturation and vessel stability, while patient-derived biomarkers, including retinal vascular imaging and elevated circulating endothelial cells (CEC), provide evidence of ongoing endothelial dysfunction in patients. These vascular abnormalities have the potential to compromise tissue perfusion, promote local hypoxia, disrupt barrier integrity and enhance inflammatory signaling, thereby exacerbating disease progression. Collectively, the evidence suggests that SMN-dependent vascular pathology may represent an under-recognized feature of SMA pathogenesis and a potential therapeutic target. Here, we review the current evidence for vascular pathology in SMA, discuss its implications for disease progression, long-term patient outcomes and therapeutic response, and consider whether vascular abnormalities are adequately addressed by existing SMN-restorative therapies.

## Introduction

The concept that spinal muscular atrophy (SMA) is a multisystem disorder is now widely accepted (Yeo and Darras, 2020). However, the contribution of non-neuronal pathologies to disease progression, and their long-term clinical significance in the era of SMN-restorative therapies, remains poorly understood. As survival and life expectancy continue to improve, understanding whether these pathologies persist, contribute to ongoing morbidity or require direct therapeutic intervention has become increasingly important.

One system that has emerged as a potentially important contributor to SMA pathogenesis is the vasculature. Endothelial cells, which form the major component of the vasculature, play fundamental roles in tissue homeostasis beyond the delivery of oxygen and nutrients. Endothelial dysfunction can impair tissue perfusion, resulting in hypoxia, but may also compromise specialized vascular barriers, alter inflammatory signaling and disrupt communication between the vasculature and surrounding tissues (Ray et al., 2023; Wang and He, 2024). The consequences of these abnormalities are likely to vary between organs according to their specialized vascular architecture and metabolic demands, with the central nervous system (CNS) being particularly vulnerable because of its high energy requirements and dependence on the integrity of the blood-CNS barriers (Fang et al., 2023).

This review will summarize the current evidence for vascular pathology in SMA and discuss its potential contribution to both neurodegeneration and systemic disease. A thorough understanding of the role of the vasculature in SMA will help identify key knowledge gaps, guide future research, and inform the development of therapeutic strategies that address the multisystemic nature of SMA.

## Spinal muscular atrophy

SMA is an inherited neuromuscular disorder affecting approximately 1 in 15,000 individuals (Belter et al., 2024). In more than 98% of cases, SMA is caused by mutations or deletions in the *survival motor neuron 1* (*SMN1*) gene, resulting in insufficient production of the essential and ubiquitously expressed survival motor neuron (SMN) protein. Low levels of SMN are also generated from the paralogous *survival motor neuron 2* (*SMN2)* gene, however, alternative splicing limits the production of full-length, functional SMN protein. These low levels of *SMN2*-derived protein permit survival while allowing SMA to manifest as a disease. Variation in *SMN2* copy number modulates SMN protein levels and is a major determinant of disease severity (reviewed by Butchbach, 2016). In SMA patients, reduced SMN expression leads to the characteristic neuromuscular phenotype of SMA, comprising lower motor neuron degeneration, neuromuscular junction disruption, skeletal muscle denervation and atrophy, culminating in progressive weakness, respiratory insufficiency and, in its most severe forms, death during early childhood (Allardyce et al., 2024; Crawford and Pardo, 1996; Hamilton and Gillingwater, 2013).

## The role of the SMN in cells and tissues

Despite its name, SMN protein is not restricted to motor neurons but is expressed in all cell types. SMN was initially characterized as an essential component of the SMN complex, which plays a central role in the biogenesis and assembly of small nuclear ribonucleoproteins (snRNPs) required for pre-mRNA splicing. However, subsequent research has established that SMN has a much broader range of cellular functions, reflecting its involvement in multiple aspects of RNA metabolism and intracellular homeostasis. These include RNA processing and splicing, mRNA transport and local translation, cytoskeletal organization, endocytosis, autophagy, ubiquitination, mitochondrial function and cellular bioenergetics (reviewed by Chaytow et al., 2018; Ottesen et al., 2023). Recent work further highlights the complexity of *SMN2* regulation and the multiple molecular mechanisms that influence the production of functional SMN protein, including cis- and trans-acting regulators of exon 7 inclusion and epigenetic control of *SMN2* expression (Virata et al., 2025).

The breadth of these functions suggests that SMN acts as an important regulator of cellular homeostasis rather than serving a single, specialized role in motor neurons. Importantly, the cellular response to SMN restoration is also not uniform. Patient-derived fibroblast studies have demonstrated substantial inter-individual variation in the response to *SMN2* splice-modifying therapies, with baseline SMN levels, *SMN2* copy number and age influencing treatment response (Signoria et al., 2024). Together, these findings highlight the complexity of SMN biology and suggest that the consequences of SMN deficiency, and its restoration, may be highly dependent on cellular and tissue context.

The requirement for SMN also varies between tissues and across the lifespan. SMN expression is highest during embryonic and early postnatal development, with a progressive decline into adulthood in both humans and mice, suggesting important developmental functions (Coovert et al., 1997; Jablonka and Sendtner, 2017; Ramos et al., 2019). While high levels are expected within the CNS, substantial expression has also been reported in peripheral organs including the heart, liver and kidneys, in a tissue- and age-dependent manner (Groen et al., 2018; Yeo and Darras, 2021). This distribution, together with the diverse cellular functions of SMN, provides a biological basis for the widespread tissue pathology observed in SMA and supports the concept that SMN deficiency may directly affect multiple cell types, including endothelial cells.

## SMA as a multisystem disorder

Accumulating evidence from patients and preclinical models has demonstrated that SMA extends beyond the neuromuscular system and should now be considered a multisystem disease. In addition to motor neuron loss and skeletal muscle atrophy, pathological changes have been described in the cardiac, vascular, musculoskeletal, immune, gastrointestinal, urinary and reproductive systems (reviewed by Hamilton and Gillingwater, 2013; Yeo and Darras, 2020; Shababi et al., 2014; Yeo and Darras, 2021). Recognizing these non-neuronal manifestations is essential to understand the full spectrum of SMA pathology and to evaluate whether current SMN-restorative therapies adequately address disease beyond the motor unit.

Of these extra-neuronal pathologies, vascular pathology has emerged as an increasingly well-characterized feature of SMA. Vascular abnormalities have been reported across multiple tissue and organ systems, suggesting that impaired vascular development and maintenance may be a fundamental consequence of SMN deficiency.

## Importance of the vascular system

The vascular system is composed of an extensive network of endothelial cell-lined blood vessels that ramify through every organ and tissue, delivering oxygen and nutrients while removing metabolic waste (Alberts et al., 2002). Beyond this fundamental role, endothelial cells and the vascular bed are highly specialized, adopting tissue-specific phenotypes that regulate vascular permeability, inflammatory cell trafficking and angiogenesis to meet the functional demands of individual organs (Mou et al., 2024). Each vascular bed therefore displays remarkable structural and functional heterogeneity, ranging from the highly restrictive blood-CNS barriers, through the continuous capillaries found in many peripheral tissues, to the fenestrated capillaries of the kidney and liver (Pluta et al., 2025). As vascular functions differ considerably between organs, the pathological consequences are highly tissue-specific (Ajoolabady et al., 2024). Understanding this vascular heterogeneity is therefore essential when considering how endothelial and vascular pathologies could contribute to the diverse manifestations of SMA.

## Vascular abnormalities may contribute to neuromuscular dysfunction in SMA

Within the neuromuscular system, which is the primary site of pathology in SMA, vascular abnormalities have been identified in the spinal cord and skeletal muscle of both preclinical models and patients, detailed in Tables 1, 2, respectively.

**Table 1:** Vascular changes in SMA preclinical mouse models.

Organ/tissue	Model	Model Timepoint	Observation	References
Neuromuscular				
Spinal cord	Taiwanese mouse	Early–late symptomatic Early symptomatic	Decreased vascular density across white and gray matter Lack of postnatal vascular development Heterogeneous distribution of BSCB protein Claudin-5 Neuronal hypoxia	(Somers et al., 2016)
	SMNΔ7 mouse	Late symptomatic	Decreased vascular density across white and gray matter Lack of postnatal vascular development	(Somers et al., 2016)
	Taiwanese mouse	Early symptomatic	Neuronal hypoxia	(Hernandez-Gerez et al., 2020)
Skeletal muscle	Taiwanese mouse	Pre–late symptomatic	Decreased vascular density across all muscle types Reduced vascular branching and network complexity Increased vessel caliber Reduced expression of PECAM-1 protein Lack of postnatal vascular development Paralleled timeline of neuromuscular degeneration	(Somers et al., 2012)
	Taiwanese mouse	Mid symptomatic	Postnatal reduction in vascular density in forelimb muscles Fewer larger intramuscular arterioles	(Somers et al., 2016)
	Taiwanese mouse	Late symptomatic	Reduced vascular pericyte coverage	(Zhou et al., 2015b)
Systemic				
Heart	Taiwanese mouse	Early symptomatic	Decreased vascular density Reduced expression of PECAM-1 protein	(Maxwell et al., 2018)
	SMNΔ7 mouse	Late symptomatic	Loss of arterial SMCs Reduced medial thickness of arterioles Decreased vascular density Cardiac oxidative stress	(Shababi et al., 2012)
Kidneys	Taiwanese mouse	Early–late symptomatic	Decreased vascular density Reduced expression of PECAM-1 protein Reduced glomerular capillary bed complexity	(Allardyce et al., 2020)
Gastrointestinal tract	Taiwanese mouse	Late symptomatic	Decreased vascular density in ileum and duodenum Reduced expression of VWF protein	(Sintusek et al., 2016)
Peripheral vasculature	Mild Taiwanese mouse	Mid symptomatic	Necrosis of ear pinnae and tails	(Hsieh-Li et al., 2000)
	Burgheron mouse	Pre symptomatic	Necrosis of ear pinnae, tails and hindlimbs	(Bogdanik et al., 2015)
	SMNΔ7 mouse	Prolonged survival due to TSA treatment Prolonged survival due to HDAC treatment Prolonged survival due to ASO treatment Prolonged survival due to scAAV9 treatment	Necrosis of ear pinnae and tails Necrosis of tails and hindlimbs Necrosis of hindlimbs Necrosis of ear pinnae and tails Necrosis of ear pinnae	(Narver et al., 2008; Riessland et al., 2010; Passini et al., 2011; Hua et al., 2011; Foust et al., 2010; Valori et al., 2010)

PM, postmortem; BSCB, blood spinal cord barrier; PECAM-1, platelet endothelial cell adhesion molecule-1; SMCs, smooth muscle cells; VWF, Von Willebrand factor; TSA, Trichostatin A; HDAC, histone deacetylase inhibitor; ASO, antisense oligonucleotide; scAAV9, self-complementary adeno-associated virus serotype 9.

**Table 2:** Clinical phenotypes of vascular deficits in SMA patients.

Organ/tissue	SMA type	Technique/procedure	Observation	References
Neuromuscular				
Spinal cord	Type I	PM tissue	Endothelial dysfunction Ultrastructural endothelial cell damage Ultrastructural cell damage to all BSCB components Functional leakage of the BSCB	(Allardyce et al., 2026)
Skeletal muscle Retina	Not reported	Skeletal muscle biopsies	Decreased vascular density	(Somers et al., 2016)
	Not reported	Skeletal muscle biopsies	Reduced vascular pericyte coverage	(Zhou et al., 2015b)
	Type II–III	OCT imaging	Decreased vascular density Reduced network complexity	(Zhou et al., 2022)
Systemic				
Peripheral vasculature	Type 0	Clinical observation	Distal digital necrosis	(Maeda et al., 2019)
	Type I	Clinical observation	Distal digital necrosis	(Araujo et al., 2009; Salmin et al., 2019; Carrasco et al., 2019)
	Type I	Skin biopsies	Necrosis of epidermis and upper dermis Thrombotic small vessel occlusion Vessel thickening Non-specific vasculitis	(Rudnik-Schöneborn et al., 2010)
	Type 0	Skin biopsies	Vasculopathy and thrombosis	(Matesanz et al., 2020)
	Type 0	Skin biopsies	Epidermal necrosis Subepidermal fibrin Dermal vessel thickening	(Weissman et al., 2021)
General endothelium	Types I–III	Blood based biomarker (CECs)	Increased occurrence of endothelial injury Decreased ability for endothelial repair	(Zhou et al., 2022)

PM, postmortem; BSCB, blood spinal cord barrier; OCT, optical coherence tomography; CECs, circulating endothelial cells.

## Spinal cord

The vascular supply of the CNS is essential for normal neuronal development, function and survival, ensuring adequate delivery of oxygen and nutrients while maintaining the highly specialized blood brain barrier (BBB) and blood spinal cord barrier (BSCB) that protect the neural microenvironment (Herculano-Houzel, 2011). As degeneration of spinal motor neurons underlies the clinical manifestation of SMA, disruption of spinal cord vascular integrity would be expected to exacerbate neuronal dysfunction and accelerate disease progression.

Recently, Allardyce et al. (2026) demonstrated important vascular and BSCB abnormalities in post-mortem tissue from patients with severe early-onset SMA. Endothelial cells, the major component of blood vessels, demonstrated significantly reduced expression of von Willebrand factor (VWF), which indicated endothelial dysfunction. Ultrastructural analyses revealed endothelial cell oedema, extensive vacuolization, and disruption of plasma membrane integrity. Importantly, pathological changes extended beyond the endothelium to other cellular components of the BSCB, with surrounding pericytes displaying vacuolization and closely apposed astrocytic end feet containing abnormal accumulation of filaments. Importantly, these structural abnormalities were accompanied by functional impairment of the BSCB, demonstrated by extravasation of fibrinogen into the spinal cord parenchyma and widespread activation of microglia, indicative of a neuroinflammatory response (Allardyce et al., 2026).

Evidence from preclinical models suggests that these vascular abnormalities arise early in disease pathogenesis. “Taiwanese” SMA mice, which model severe disease, show a decreased vascular density throughout both the gray and white matter of the spinal cord before the onset of neuromuscular symptoms (Somers et al., 2016). Vascular deficits progressively worsened with disease progression and were associated with increased neuronal hypoxia, indicating impaired tissue perfusion. In addition, a subsequent study found that NSC34 neurons with low levels of SMN protein were significantly more likely to die when subjected to a hypoxic environment (Hernandez-Gerez et al., 2020). Furthermore, altered expression of the tight junction protein claudin-5 suggested compromised BSCB integrity (Somers et al., 2016). This presence of vascular abnormalities before symptom onset suggests that vascular dysfunction is likely to contribute to, rather than result from, motor neuron degeneration.

Taken together, the evidence suggests that endothelial dysfunction and BSCB disruption may be integral features of SMA pathology within the spinal cord. By impairing tissue perfusion, promoting neuronal hypoxia and permitting entry of circulating inflammatory mediators into the CNS, these abnormalities are likely to establish a hypoxic and neuroinflammatory microenvironment that accelerates motor neuron dysfunction and degeneration.

## Skeletal muscle

At the distal end of the motor unit, the skeletal muscle microvasculature plays a critical role in delivering oxygen and nutrients required to support muscle metabolism, maintenance and repair (Kusters and Barrett, 2015).

In skeletal muscle from both severe (Taiwanese) and milder (SMNΔ7) SMA mouse models, capillary density was significantly reduced and accompanied by decreased vascular branching, reduced network complexity and increased vessel caliber. These abnormalities were observed across both fast- and slow-twitch muscles, irrespective of the degree of neuromuscular junction denervation, suggesting that vascular pathology develops independently of motor neuron loss. Although absent at birth, these vascular defects emerged during early postnatal development, but preceded neuromuscular pathology (Somers et al., 2012, 2016). In addition, supporting components of the vasculature were also affected, with significant reductions in vascular pericyte coverage observed in both SMA mice and patient muscle biopsies (Zhou et al., 2015b). Importantly, these findings were validated in historical skeletal muscle biopsies from patients with Type I and Type II SMA. Here, a significant reduction in the capillary-to-muscle fiber ratio, a widely accepted measure of muscle vascularization, was observed. Furthermore, this declined approximately tenfold between birth and 3 years of age (Somers et al., 2016). This progressive loss of muscle vascularization suggests that vascular pathology is likely to proceed throughout disease progression rather than representing a developmental defect alone.

These findings suggest that impaired angiogenesis and defective vascular maturation are significant features of skeletal muscle pathology in SMA. Reduced capillary density and disruption of the supporting microvasculature are likely to impair tissue perfusion, limiting oxygen and nutrient delivery and compromising the metabolic support required for muscle maintenance and repair (Mukund and Subramaniam, 2019). These vascular abnormalities have the potential therefore, to contribute directly to muscle dysfunction, independent of motor neuron degeneration.

## Vascular pathology extends beyond the neuromuscular system

Beyond the neuromuscular system, vascular abnormalities are reported in multiple systemic organs, detailed in Tables 1, 2, respectively.

## Heart

Cardiovascular abnormalities represent one of the most common non-neuromuscular comorbidities, with reports of congenital cardiac defects (including atrial and ventricular septal defects) and hypoplastic left heart syndrome being consistently reported in patients with SMA (Møller et al., 1990; Rudnik-Schöneborn et al., 2008; Wijngaarde et al., 2017). This has led to medical insurance claims among SMA patients in the United States most commonly citing cardiovascular abnormalities (Lipnick et al., 2019).

Preclinical studies have identified pronounced vascular abnormalities in the myocardium of the Taiwanese and SMNΔ7 mouse models. Here, capillary density was significantly reduced at early symptomatic stages of disease, demonstrated by reduced expression of the endothelial markers platelet endothelial cell adhesion molecule-1 (PECAM-1) and VWF, and associated oxidative stress (Maxwell et al., 2018). In a separate study, coronary arteries show a loss of smooth muscle cells in the tunica media associated with a pre-symptomatic decrease in arterial wall thickness and remodeling, however endothelial cell integrity appeared unaffected as demonstrated by levels of endothelial nitric oxide synthase (e-NOS) (Shababi et al., 2012). In this study, the systemic restoration of SMN using self-complementary AAV9 gene therapy only partially rescued coronary artery morphology, improving arterial wall thickness, however, myocardial capillary density remained significantly reduced (Shababi et al., 2012). This incomplete rescue of cardiac microvasculature following SMN-restorative therapy raises the possibility that vascular abnormalities may persist despite correction of SMN deficiency by gene therapy. Reduced myocardial capillary density would be expected to impair perfusion and compromise cardiac function (Giacobbe et al., 2026). Consistent with this, echocardiographic assessment of the milder *Smn2B/–* SMA mouse model demonstrated increased systolic function and global longitudinal strain, indicative of elevated cardiac workload and physiological stress (Nair et al., 2025).

## Kidneys

The renal vasculature is characterized by specialized fenestrated capillaries that form the selective filtration barrier required to maintain systemic homeostasis (Mou et al., 2024). In severe Taiwanese SMA mouse models, the decreased expression of PECAM-1 indicated a marked reduction in renal vascular density (Allardyce et al., 2020). This reduction was particularly pronounced within the renal cortex, the primary site of glomerular filtration, and was accompanied by reduced capillary network complexity within nephrons. This would be predicted to impair glomerular filtration and overall renal function. Consistent with these findings, alterations in renal biomarkers have been reported in SMA patients, including changes in serum creatinine, cystatin C, sodium, glucose and calcium concentrations, supporting the presence of clinically relevant renal involvement in SMA (Nery et al., 2019). These findings suggest that vascular dysfunction has the potential to impair renal function through disruption of the specialized microvascular network required for filtration.

## Gastrointestinal tract

The gastrointestinal tract is a highly vascularized organ, receiving approximately 20% of resting cardiac output to support digestion and nutrient absorption (Granger et al., 2015). In severe SMA mouse models, capillary density was significantly reduced in the duodenum and ileum and was accompanied by increased enteric neuronal density and macrophage infiltration (Sintusek et al., 2016). Gastrointestinal dysfunction is common in SMA patients and is often attributed to impaired smooth and skeletal muscle function (Corsello et al., 2021). However, impaired intestinal perfusion has the potential to contribute to these symptoms. Once again, systemic restoration of SMN using antisense oligonucleotide (ASO) therapy only partially restored intestinal vascular density in preclinical models (Sintusek et al., 2016). Furthermore, gastrointestinal complications continue to be reported in patients receiving SMN-targeted therapies (Yu and Liu, 2024; Zhang et al., 2024; Zhong et al., 2023).

## Spleen

The spleen is a highly vascularized lymphoid organ that plays essential roles in immune surveillance, blood filtration and erythrocyte turnover (Lewis et al., 2019). Unlike other peripheral organs, histological analyses revealed no significant differences in vascular architecture or vessel density of the spleen (Thomson et al., 2017). However, functional vascular deficits have been reported, with SMA mouse models demonstrating reduced splenic perfusion, diffuse hypoxia and decreased peak systolic flow velocity in the splenic artery (Deguise et al., 2020; Hernandez-Gerez et al., 2020). As splenic growth and development are dependent on an adequate blood supply, perfusion deficits could contribute to the reduced spleen size observed in SMA models and the gross pathologies observed in patient spleens (Thomson et al., 2017).

## Peripheral vasculature

In the peripheral circulation, distal digital necrosis has been reported in severe patient cases, both in conjunction with, and independently of congenital heart malformations (Araujo et al., 2009; Carrasco et al., 2019; Maeda et al., 2019; Matesanz et al., 2020; Rudnik-Schöneborn et al., 2010; Salmin et al., 2019; Weissman et al., 2021). At the ultrastructural level, skin from these digits showed extensive necrosis of the dermis and epidermis, with thrombotic occlusion of small blood vessels, vascular wall thickening, inflammatory infiltration of the dermal papillae and features of vasculitis, which together are consistent with impaired tissue perfusion and vascular remodeling (Matesanz et al., 2020; Rudnik-Schöneborn et al., 2010; Weissman et al., 2021). In parallel, distal necrosis affecting the ears, tail and hindlimbs were among the first vascular phenotypes described in SMA mouse models. In the Taiwanese and Burgheron mouse models, distal necrosis was observed predominantly in milder phenotypes with prolonged survival, suggesting that peripheral vascular pathology develops as disease progresses (Bogdanik et al., 2015; Hsieh-Li et al., 2000).

Together, these findings demonstrate that vascular pathology in SMA extends well beyond the neuromuscular system and affects multiple peripheral organs and tissues (Figure 1). Although the manifestations differ between tissues, a consistent pattern emerges of impaired vascular development, aberrant microvascular density, and disruption of specialized vascular functions. Such abnormalities have the ability to compromise tissue homeostasis and contribute to organ-specific pathology, supporting the idea that vascular pathology is most likely to be a direct consequence of SMN deficiency rather than a secondary effect of motor neuron degeneration.

**Figure 1:**
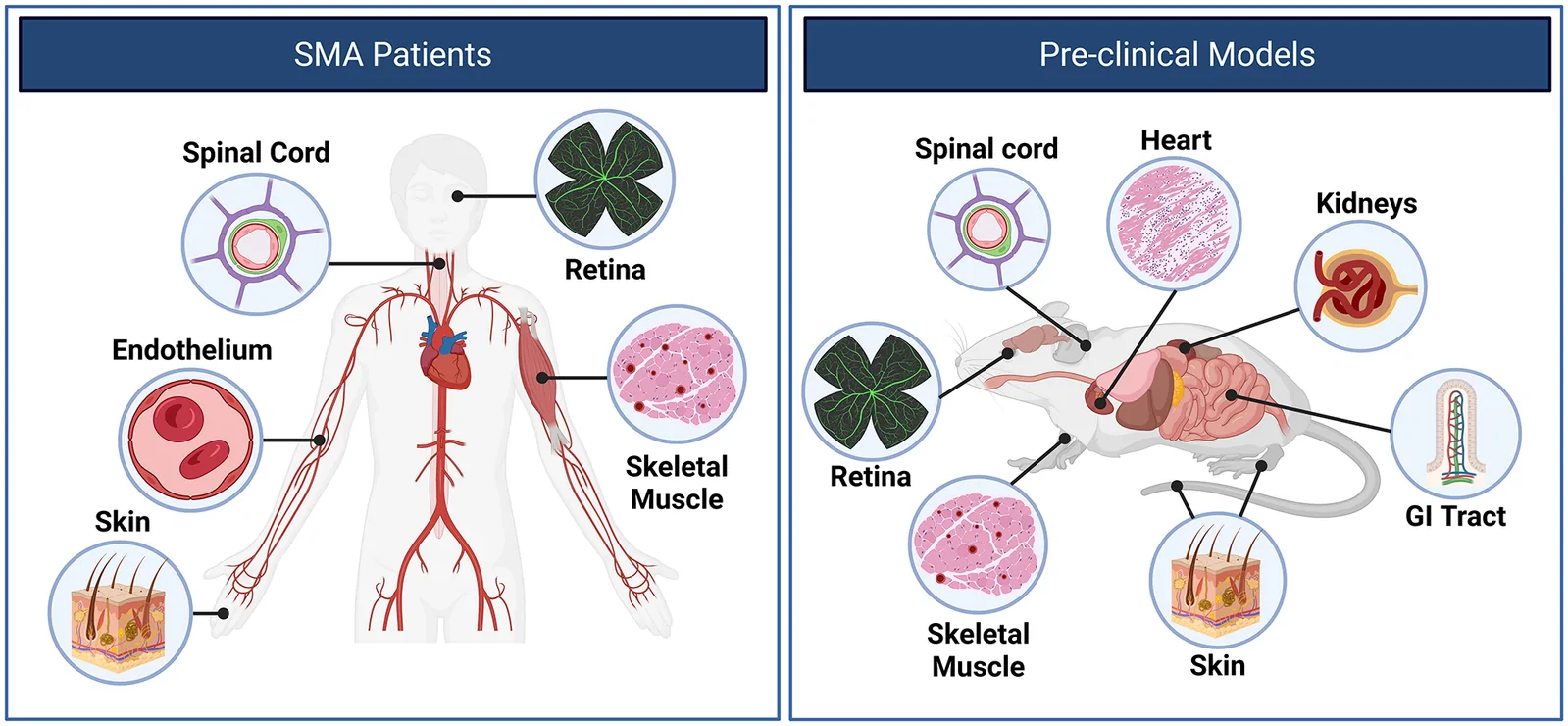
Sites of vascular pathology in SMA patients and preclinical models. Created in BioRender. (Allardyce et al., 2026) https://BioRender.com/ja0zg7b.

## SMN depletion directly impairs endothelial cell function

Experimental studies using isolated endothelial cells have demonstrated that SMN depletion directly impairs endothelial function, supporting the hypothesis that vascular pathology arises, at least in part, through cell-autonomous mechanisms. Using both SMN-depleted human umbilical vein endothelial cells (HUVECs) and aortic endothelial cells isolated from severe Taiwanese SMA mice, Zhou et al. (2022) demonstrated impaired endothelial cell migration and network formation, characterized by reduced tube formation and vessel branching. As endothelial migration and tube formation are fundamental processes in angiogenesis (Lamalice et al., 2007), these findings provide a plausible potential mechanistic explanation for the impaired vascular development in SMA. Although the molecular pathways linking SMN deficiency to endothelial dysfunction remain incompletely understood, these studies provide compelling evidence that endothelial dysfunction is a direct consequence of SMN depletion. Further investigation into the mechanisms underlying endothelial dysfunction will be essential to understand how vascular pathology develops and to identify therapeutic strategies to preserve vascular health.

## Biomarkers of vascular damage suggest ongoing pathology in SMA patients

While evidence from experimental models and post-mortem studies have established our current understanding of vascular pathology in SMA, it does not allow us to assess the extent or progression of these abnormalities in patients living with SMA. The identification of non-invasive biomarkers capable of detecting ongoing vascular injury is essential to establish the clinical relevance of vascular pathology and to monitor vascular health following therapeutic intervention.

## Optical coherence tomography

Optical coherence tomography (OCT) is a non-invasive imaging method that enables visualization of the retinal vasculature (Zeppieri et al., 2023). As an extension of the CNS, the retina provides a valuable surrogate for assessing CNS vascular health (Kashani et al., 2021). In patients with Type II and III SMA, OCT revealed significant reductions in retinal vascular density and network complexity compared with age-matched controls (Zhou et al., 2022). As alterations in the retinal vasculature are widely recognized as biomarkers of cerebrovascular pathology in neurological disease (Mainster, 1990), these findings provide *in vivo* evidence that vascular abnormalities are present in patients living with SMA.

Studies in the Taiwanese SMA mouse model provide mechanistic insight into these clinical observations. The primary retinal vascular network forms postnatally in mice, providing a unique opportunity to study developmental angiogenesis (Stahl et al., 2010). Zhou et al. (2022) demonstrated delayed retinal vascular development, characterized by reduced radial outgrowth of the vascular network from the optic nerve, decreased vessel complexity and reduced smooth muscle cell coverage, indicative of impaired angiogenesis and defective vessel maturation. Once again, these abnormalities preceded the onset of neuromuscular pathology and suggest that vascular dysfunction is an independent and early pathological event.

## Circulating endothelial cells

Under physiological conditions, endothelial cells remain tightly attached to the vessel wall, with few detaching to become circulating endothelial cells (CEC). However, endothelial injury promotes their detachment, with increased CEC numbers representing an established biomarker of vascular damage (Erdbruegger et al., 2006). Patients with SMA (Types I–III) have significantly increased CEC numbers, which are correlated with clinical disease severity. Furthermore, SMA patients also demonstrate a reduced capacity for endothelial repair, indicated by decreased numbers of functional endothelial progenitor cell, assessed by their ability to generate colony-forming units (Zhou et al., 2022). Together, this indicates that vascular homeostasis is tipping toward injury (Deanfield et al., 2007).

These findings demonstrate that vascular abnormalities are not confined to experimental models or end-stage disease but can be detected in patients living with SMA. The continued development and validation of vascular biomarkers will be essential to define and monitor vascular involvement in the post-therapeutic landscape.

## Implications: Could microvascular dysfunction exacerbate neurodegeneration in SMA?

Vascular dysfunction has the potential to exacerbate neurodegeneration in SMA through at least two complementary mechanisms: impaired tissue oxygenation and disruption of the BSCB.

## Impaired tissue oxygenation

Given the exceptionally high metabolic demands of the CNS, even modest reductions in tissue perfusion have the potential to promote neuronal dysfunction and degeneration. Indeed, the CNS consumes approximately 20% of the body's resting oxygen despite accounting for only a small proportion of total body mass, making it particularly susceptible to hypoxic injury (Eltzschig and Carmeliet, 2011; Hwang and Lee, 2011; Silver and Erecitiska, 1998).

The abnormal vascular architecture, loss of key endothelial proteins including PECAM-1 and VWF, and ultrastructural endothelial damage described in the spinal cord of SMA patients and preclinical models, are ideally positioned to compromise tissue perfusion and oxygen delivery. Consistent with this, an increased number of hypoxic neurons are present in the brain and spinal cord of early symptomatic SMA mouse models, alongside evidence of hypoxia in peripheral tissues including skeletal muscle, kidney, spleen and liver (Hernandez-Gerez et al., 2020; Somers et al., 2016). Evidence consistent with hypoxia have also been reported in patient autopsy tissue, where markers of oxidative stress were identified in the brain and spinal motor neurons of patients with Type I SMA (Hayashi et al., 2002). Although motor neurons are inherently sensitive to oxygen deprivation, SMN-deficient neurons display an even greater susceptibility to hypoxic stress, resulting in metabolic dysfunction, cell-cycle arrest and ultimately cell death (Hernandez-Gerez et al., 2020; Watzlawik et al., 2015). Hypoxia, through vascular dysfunction, may therefore represent an additional pathological insult capable of amplifying neuronal vulnerability in SMA and accelerating neurodegeneration driven directly by SMN depletion in motor neurons.

## Disruption of the BSCB

In addition to impaired oxygen delivery, vascular pathology may exacerbate neurodegeneration through disruption of the BSCB. The BSCB is essential for maintaining a specialized neural microenvironment by regulating the entry of circulating molecules, inflammatory mediators and immune cells into the spinal cord (Chopra et al., 2021). Loss of barrier integrity therefore has the potential to expose vulnerable motor neurons to neurotoxic blood-derived components and promote chronic neuroinflammation (Jin et al., 2021; Winkler et al., 2011).

Post-mortem analysis of the spinal cord from patients with severe SMA demonstrated ultrastructural damage to all cellular components (endothelial cells, basement membrane, pericytes and astrocytes) of the BSCB, which was associated with functional impairment of the BSCB demonstrated by extravasation of fibrinogen into the spinal cord parenchyma, accompanied by widespread microglial activation (Allardyce et al., 2026). Clinical pharmacokinetic studies demonstrated rapid appearance of intrathecally administered nusinersen in the systemic circulation following cerebrospinal fluid injection, suggesting increased leakage across the BSCB (Chiriboga et al., 2016). Similarly, elevated cerebrospinal fluid total protein and albumin quotient (QAlb), which are established clinical indicators of barrier dysfunction, have been reported in both adult and pediatric SMA patients receiving nusinersen (Müschen et al., 2021; Wurster et al., 2019).

Collectively, these findings suggest that disruption of the BSCB may represent an additional mechanism through which vascular pathology could further accelerate neurodegeneration in SMA by exposing the spinal cord to circulating blood-derived molecules and promoting neuroinflammation.

## Implications: Could vascular pathology contribute to long-term systemic complications in SMA?

Over 10 years ago, a review posed the question: “Does ameliorating disease progression by targeting motor neurons lead to the ‘uncovering' of less severe multisystem phenotypes?” (Hamilton and Gillingwater, 2013). The widespread vascular abnormalities described here provide further support for this concept. Historically, severe SMA was associated with a limited life expectancy, meaning that patients often did not survive long enough for chronic, multisystem pathologies to become clinically apparent. However, the availability of SMN-restorative therapies has dramatically improved survival (Yu et al., 2026), bringing greater attention to the long-term consequences of multisystem pathology.

Evidence from preclinical therapeutic studies suggests that prolonged survival may itself reveal previously subclinical vascular pathologies. Prior to the availability of current SMN-restorative therapies, several experimental approaches that modestly extended lifespan, resulted in distal necrosis that affected the ears, tail and hindlimbs (Foust et al., 2010; Hua et al., 2011; Narver et al., 2008; Passini et al., 2011; Riessland et al., 2010). In contrast, systemic restoration of SMN using ASOs or self-complementary AAV9 gene therapy delayed or prevented the development of digital necrosis, particularly when administered pre-symptomatically (Bogdanik et al., 2015; Hua et al., 2010, 2011; Zhou et al., 2013). These findings suggest that vascular pathology may emerge as survival is prolonged, but also that it could be targeted by systemic upregulation of SMN protein.

The heart, kidneys, liver and gastrointestinal tract are all highly vascularized organs that depend on specialized microvascular networks to maintain tissue homeostasis (Mou et al., 2024). Experimental studies have demonstrated vascular abnormalities affecting each of these organs, including reduced capillary density, impaired vascular remodeling and altered tissue perfusion. Although the long-term clinical consequences of these abnormalities remain unknown, persistent vascular dysfunction could compromise organ function over time, particularly as patients age.

Importantly, several experimental studies reviewed here suggest that aspects of vascular pathology are only partially rescued following SMN-restorative therapy, and that this may be dependent upon the route and mechanisms of delivery. Whether residual vascular abnormalities contribute to chronic cardiovascular, renal or gastrointestinal dysfunction in treated individuals remains to be determined. Longitudinal studies incorporating vascular biomarkers and functional assessment of peripheral organs will therefore be essential to establish the long-term clinical significance of vascular pathology in SMA.

## Do current SMN-restorative therapies adequately target vascular pathology?

The introduction of SMN-restorative therapies has transformed the clinical management of SMA, dramatically improving survival, motor function and quality of life (Yu et al., 2026). However, all four currently approved therapies were developed to restore SMN levels in motor neurons, thereby halting neuronal loss. Whether these therapies also restore SMN expression in extra neuronal tissues, including the vasculature, and reverse vascular pathology remains largely unknown.

The first approved therapy, nusinersen (Spinraza; Biogen, Cambridge, MA), is an ASO administered intrathecally to increase production of full-length SMN protein from the *SMN2* gene (Neil and Bisaccia, 2019). Due to its route of administration, nusinersen is largely confined to the CNS and should have limited access to peripheral tissues. While there is evidence for leakage of nusinersen through the BSCB into the systemic circulation, it is rapidly metabolized, excreted and therefore not biologically active (Chiriboga et al., 2016). Although this strategy effectively targets spinal motor neurons, it is unlikely to directly address endothelial and vascular pathology in systemic organs. Supporting this concept, preclinical studies using analogous ASOs demonstrated greater improvements in survival when treatment was delivered both systemically and to the CNS than when administered to the CNS alone, suggesting that correction of peripheral pathology contributes to therapeutic benefit and additional systemic delivery may be beneficial (Hua et al., 2011, 2015; Zhou et al., 2015a).

In contrast, the gene replacement therapy onasemnogene abeparvovec (Zolgensma; AveXis/Novartis, Chicago, IL) which delivers a functional *SMN1* gene systemically, allowing widespread distribution throughout the body while also crossing the BBB and BSCB via the AAV9 vector (Bitetti et al., 2022). In principle, this broader biodistribution offers the potential to target both central and peripheral pathology (Reilly et al., 2022). However, despite its ability to access the CNS, AAV9 is a relatively poor transducer of endothelial cells, limiting transgene delivery to the vasculature and possibly therapeutic effect (Merkel et al., 2017). Consistent with this, experimental studies demonstrated only partial rescue of vascular pathology following systemic SMN restoration with AAV9 (Shababi et al., 2012).

Among currently available therapies, risdiplam (Evrysdi; Roche, Basel, Switzerland) may offer the greatest potential to address vascular pathology. As an orally administered small-molecule *SMN2* splicing modifier, risdiplam readily distributes throughout both the CNS and peripheral tissues and enters cells by passive diffusion (Fowler et al., 2022; Poirier et al., 2018). Clinical studies have demonstrated sustained increases in SMN protein levels in peripheral blood, suggesting broad tissue exposure (Messina and Sframeli, 2020). Nevertheless, whether this widespread biodistribution translates into restoration of endothelial function or reversal of vascular pathology remains unknown, and long-term vascular outcomes have yet to be investigated (Singh et al., 2020).

Overall, SMN-restorative therapies have revolutionized the treatment of SMA, yet their ability to correct vascular pathology remains uncertain. Given the evidence that endothelial dysfunction is widespread, detectable in living patients and may contribute to both neurodegeneration and systemic disease, evaluation of vascular outcomes should become an important consideration in future preclinical and clinical studies. Incorporating vascular biomarkers alongside conventional neurological outcome measures will be essential to determine whether restoration of SMN is sufficient to normalize vascular health or whether the vasculature represents an additional therapeutic target in SMA.

## Future perspectives

Although considerable progress has been made in defining the vascular phenotype in SMA, important questions remain regarding clinical significance, underlying mechanisms, and therapeutic implications.

A priority for future research will be translating these findings into clinical practice. Further development and validation of non-invasive vascular biomarkers could provide valuable tools for monitoring vascular health throughout disease progression and assessing vascular responses to SMN-restorative therapies. Longitudinal studies in treated patients will be particularly important to determine whether vascular abnormalities persist, progress or are reversed following each of the currently approved therapies. Together, these approaches could establish the clinical significance of vascular pathology and determine whether it is adequately addressed by current therapies.

In addition, it remains important to define the molecular pathways through which SMN deficiency disrupts endothelial cell function and to establish how these changes affect vascular development, maintenance and barrier integrity across different organ systems. Several complementary approaches could address these questions: transcriptomic analyses and single-cell RNA sequencing to identify disease-associated changes in endothelial gene expression and cellular pathways; endothelial-specific SMN depletion models to establish a direct link between SMN depletion and vascular defects; advanced human *in vitro* models, (organoids, assembloids and multicellular co-culture systems) to provide more physiologically relevant platforms for study. These approaches will guide us toward a more complete understanding of the role of vascular pathology in SMA.

Finally, if vascular abnormalities persist despite SMN-restorative therapy, a careful study of the route and mechanisms by which SMN levels are restored may lead to a better understanding of how to target the vascular system. Equally, combinatorial approaches may provide an opportunity to address residual pathology and further improve long-term outcomes for individuals living with SMA.

## Conclusion

The evidence reviewed here demonstrates that vascular pathology is a widespread feature of SMA, affecting both the CNS and multiple peripheral organs. Rather than representing a secondary consequence of motor neuron degeneration, accumulating evidence suggests that endothelial dysfunction and resulting vascular pathology is an intrinsic consequence of SMN deficiency, resulting in impaired angiogenesis, altered vascular architecture and compromised barrier integrity. Importantly, these abnormalities are detectable in patients living with SMA and have the potential to contribute to both neurodegeneration and long-term systemic pathology.

The remarkable success of SMN-restorative therapies has fundamentally changed the outlook for individuals living with SMA. However, whether these treatments fully restore vascular health remains uncertain. As survival continues to improve, understanding the long-term consequences of vascular dysfunction and determining whether it requires direct therapeutic intervention will become increasingly important. The vasculature deserves to be considered alongside motor neurons as a potentially important contributor to SMA pathogenesis and a target for future therapeutic development.

## References

[B1] Ajoolabady A. Pratico D. Ren J. (2024). Endothelial dysfunction: mechanisms and contribution to diseases. Acta Pharmacol. Sin. 45:20232031. doi: 10.1038/s41401-024-01295-8 PMC11420364 38773228

[B2] Alberts B. Johnson A. Lewis J. Raff M. Roberts K. Walter P. (2002). Blood vessels and endothelial cells. Available online at: https://www.ncbi.nlm.nih.gov/books/NBK26848/ (Accessed May 12, 2026).

[B3] Allardyce H. Kuhn D. Hernandez-Gerez E. Hensel N. Huang Y. T. Faller K. . (2020). Renal pathology in a mouse model of severe spinal muscular atrophy is associated with downregulation of glial cell-line derived neurotrophic factor (GDNF). Hum. Mol. Genet. 29, 2365–2378. doi: 10.1093/hmg/ddaa126 32588893

[B4] Allardyce H. Lanz H. Lawrence B. D. Crawford T. O. Sumner C. J. Parson S. H. (2026). Microvascular pathology in the spinal cord of severe spinal muscular atrophy patients. Acta Neuropathol. Commun. 14:65. doi: 10.1186/s40478-026-02232-y PMC13011495 41692783

[B5] Allardyce H. Lawrence B. D. Crawford T. O. Sumner C. J. Parson S. H. (2024). A reassessment of spinal cord pathology in severe infantile spinal muscular atrophy. Neuropathol. Appl. Neurobiol. 50:e13013. doi: 10.1111/nan.13013 PMC11972064 39449271

[B6] Araujo A. de Prufer Q. C. Araujo M. Swoboda K. J. (2009). Vascular perfusion abnormalities in infants with spinal muscular atrophy. J. Pediatr. 155, 292–294. doi: 10.1016/j.jpeds.2009.01.071 PMC3250227 19619755

[B7] Belter L. Taylor J. L. Jorgensen E. Glascock J. Whitmire S. M. Tingey J. J. . (2024). Newborn screening and birth prevalence for spinal muscular atrophy in the US. JAMA Pediatr. 178, 946–949. doi: 10.1001/jamapediatrics.2024.1911 PMC11250364 39008286

[B8] Bitetti I. Lanzara V. Margiotta G. Varone A. (2022). Onasemnogene abeparvovec gene replacement therapy for the treatment of spinal muscular atrophy: a real-world observational study. Gene Ther. 30:592. doi: 10.1038/s41434-022-00341-6 PMC10457192 35606491

[B9] Bogdanik L. P. Osborne M. A. Davis C. Martin W. P. Austin A. Rigo F. . (2015). Systemic, postsymptomatic antisense oligonucleotide rescues motor unit maturation delay in a new mouse model for type II/III spinal muscular atrophy. Proc. Natl. Acad. Sci. USA. 112, E5863–E5872. doi: 10.1073/pnas.1509758112 PMC4629342 26460027

[B10] Butchbach M. E. R. (2016). Copy number variations in the survival motor neuron genes: implications for spinal muscular atrophy and other neurodegenerative diseases. Front. Mol. Biosci. 3:181888. doi: 10.3389/fmolb.2016.00007 PMC4785180 27014701

[B11] Carrasco D. Magoulas P. Scull J. C. Jarrell J. A. Lalani S. R. Wangler M. F. (2019). Digital necrosis in an infant with severe spinal muscular atrophy. Neurol. Genet. 5:e361. doi: 10.1212/NXG.0000000000000361 PMC6807655 31742229

[B12] Chaytow H. Huang Y. T. Gillingwater T. H. Faller K. M. E. (2018). The role of survival motor neuron protein (SMN) in protein homeostasis. Cell. Mol. Life Sci. 75, 3877–3894. doi: 10.1007/s00018-018-2849-1 PMC6182345 29872871

[B13] Chiriboga C. A. Swoboda K. J. Darras B. T. Iannaccone S. T. Montes J. De Vivo D. C. . (2016). Results from a phase 1 study of nusinersen (ISIS-SMN Rx) in children with spinal muscular atrophy. Neurology 86, 890–897. doi: 10.1212/WNL.0000000000002445 PMC4782111 26865511

[B14] Chopra N. Menounos S. Choi J. P. Hansbro P. M. Diwan A. D. Das A. (2021). Blood-spinal cord barrier: its role in spinal disorders and emerging therapeutic strategies. NeuroSci 3:1. doi: 10.3390/neurosci3010001 PMC11523733 39484675

[B15] Coovert D. D. Le T. T. McAndrew P. E. Strasswimmer J. Crawford T. O. Mendell J. R. . (1997). The survival motor neuron protein in spinal muscular atrophy. Hum. Mol. Genet. 6, 1205–1214. doi: 10.1093/hmg/6.8.1205 9259265

[B16] Corsello A. Scatigno L. Pascuzzi M. C. Calcaterra V. Dilillo D. Vizzuso S. . (2021). Nutritional, gastrointestinal and endo-metabolic challenges in the management of children with spinal muscular atrophy type 1. Nutrients 13:2400. doi: 10.3390/nu13072400 PMC8308588 34371910

[B17] Crawford T. O. Pardo C. A. (1996). “The neurobiology of childhood spinal muscular atrophy,” in Neurobiology of Disease, Vol. 3, No. 2 (San Diego, CA: Academic Press Inc.), 97–110. doi: 10.1006/nbdi.1996.0010 9173917

[B18] Deanfield J. E. Halcox J. P. Rabelink T. J. (2007). Endothelial function and dysfunction: testing and clinical relevance. Circulation 115, 1285–1295. doi: 10.1161/CIRCULATIONAHA.106.652859 17353456

[B19] Deguise M. O. Beauvais A. Schneider B. L. Kothary R. (2020). Blood flow to the spleen is altered in a mouse model of spinal muscular atrophy. J. Neuromuscl. Dis. 7, 315–322. doi: 10.3233/JND-200493 32333548

[B20] Eltzschig H. K. Carmeliet P. (2011). Hypoxia and inflammation. N. Engl. J. Med. 364, 656–665. doi: 10.1056/NEJMra0910283 PMC3930928 21323543

[B21] Erdbruegger U. Haubitz M. Woywodt A. (2006). Circulating endothelial cells: a novel marker of endothelial damage. Clin. Chim. Acta 373, 17–26. doi: 10.1016/j.cca.2006.05.016 16836991

[B22] Fang Y. Hsieh Y. Hu C. (2023). Endothelial dysfunction in neurodegenerative diseases. Int. J. Mol. Sci. 24, 1–27. doi: 10.3390/ijms24032909 PMC9918222 36769234

[B23] Foust K. D. Wang X. McGovern V. L. Braun L. Bevan A. K. Haidet A. M. . (2010). Rescue of the spinal muscular atrophy phenotype in a mouse model by early postnatal delivery of SMN. Nat. Biotechnol. 28, 271–274. doi: 10.1038/nbt.1610 PMC2889698 20190738

[B24] Fowler S. Brink A. Cleary Y. Günther A. Heinig K. Husser C. . (2022). “Addressing today's absorption, distribution, metabolism, and excretion (ADME) challenges in the translation of in vitro ADME characteristics to humans: a case study of the SMN2 mRNA splicing modifier risdiplam,” in Drug Metabolism and Disposition, Vol. 50, No. 1 (Rockville, MD: American Society for Pharmacology and Experimental Therapy), 65–75. doi: 10.1124/dmd.121.000563 34620695

[B25] Giacobbe F. Meynet P. Balducci M. Capoccia S. Cimino R. A. Morena A. . (2026). Coronary microvascular dysfunction in cardiomyopathies: insights on clinical and prognostic roles. Rev. Cardiovasc. Med. 27:46829. doi: 10.31083/RCM46829 PMC12959992 41789319

[B26] Granger D. N. Holm L. Kvietys P. (2015). The gastrointestinal circulation: physiology and pathophysiology. Compr. Physiol. 5, 1541–1583. doi: 10.1002/j.2040-4603.2015.tb00651.x 26140727

[B27] Groen E. J. N. Perenthaler E. Courtney N. L. Jordan C. Y. Shorrock H. K. van der Hoorn D. . (2018). Temporal and tissue-specific variability of SMN protein levels in mouse models of spinal muscular atrophy. Hum. Mol. Genet. 27, 2851–2862. doi: 10.1093/hmg/ddy195 PMC6077828 29790918

[B28] Hamilton G. Gillingwater T. H. (2013). Spinal muscular atrophy: going beyond the motor neuron. Trends Mol. Med. 19, 40–50. doi: 10.1016/j.molmed.2012.11.002 23228902

[B29] Hayashi M. Araki S. Arai N. Kumada S. Itoh M. Tamagawa K. . (2002). Oxidative stress and disturbed glutamate transport in spinal muscular atrophy. Brain Dev. 24, 770–775. doi: 10.1016/S0387-7604(02)00103-1 12453601

[B30] Herculano-Houzel S. (2011). Scaling of brain metabolism with a fixed energy budget per neuron: implications for neuronal activity, plasticity and evolution. PLoS ONE 6:e17514. doi: 10.1371/journal.pone.0017514 PMC3046985 21390261

[B31] Hernandez-Gerez E. Dall'Angelo S. Collinson J. M. Fleming I. N. Parson S. H. (2020). Widespread tissue hypoxia dysregulates cell and metabolic pathways in SMA. Ann. Clin. Transl. Neurol. 7, 1580–1593. doi: 10.1002/acn3.51134 PMC7480929 32790171

[B32] Hsieh-Li H. M. Chang J. G. Jong Y. J. Wu M. H. Wang N. M. Tsai C. H. . (2000). A mouse model for spinal muscular atrophy. Nat. Genet. 24, 66–70. doi: 10.1038/71709 10615130

[B33] Hua Y. Liu Y. H. Sahashi K. Rigo F. Frank Bennett C. Krainer A. R. (2015). Motor neuron cell-nonautonomous rescue of spinal muscular atrophy phenotypes in mild and severe transgenic mouse models. Genes Dev. 29, 288–297. doi: 10.1101/gad.256644.114 PMC4318145 25583329

[B34] Hua Y. Sahashi K. Hung G. Rigo F. Passini M. A. Bennett C. F. . (2010). Antisense correction of SMN2 splicing in the CNS rescues necrosis in a type III SMA mouse model. Genes Dev. 24, 1634–1644. doi: 10.1101/gad.1941310 PMC2912561 20624852

[B35] Hua Y. Sahashi K. Rigo F. Hung G. Horev G. Bennett C. F. . (2011). Peripheral SMN restoration is essential for long-term rescue of a severe spinal muscular atrophy mouse model. Nature 478, 123–126. doi: 10.1038/nature10485 PMC3191865 21979052

[B36] Hwang A. B. Lee S. J. (2011). Regulation of life span by mitochondrial respiration: the HIF-1 and ROS connection. Aging 3:304. doi: 10.18632/aging.100292 PMC3091523 21389351

[B37] Jablonka S. Sendtner M. (2017). Developmental regulation of SMN expression: pathophysiological implications and perspectives for therapy development in spinal muscular atrophy. Gene Therapy 24, 506–513. doi: 10.1038/gt.2017.46 28556834

[B38] Jin L. Y. Li J. Wang K. F. Xia W. W. Zhu Z. Q. Wang C. R. . (2021). Blood-spinal cord barrier in spinal cord injury: a review. J. Neurotrauma 38, 1203–1224. doi: 10.1089/neu.2020.7413 33292072

[B39] Kashani A. H. Asanad S. Chan J. W. Singer M. B. Zhang J. Sharifi M. . (2021). Past, present and future role of retinal imaging in neurodegenerative disease. Prog. Retin. Eye Res. 83:100938. doi: 10.1016/j.preteyeres.2020.100938 PMC8280255 33460813

[B40] Kusters Y. H. A. M. Barrett E. J. (2015). Muscle microvasculature's structural and functional specializations facilitate muscle metabolism. Am. J. Physiol. Endocrinol. Metab. 310:E379. doi: 10.1152/ajpendo.00443.2015 PMC4888529 26714849

[B41] Lamalice L. Le Boeuf F. Huot J. (2007). Endothelial cell migration during angiogenesis. Circ. Res. 100, 782–794. doi: 10.1161/01.RES.0000259593.07661.1e 17395884

[B42] Lewis S. M. Williams A. Eisenbarth S. C. (2019). Structure and function of the immune system in the spleen. Sci. Immunol. 4:eaau6085. doi: 10.1126/sciimmunol.aau6085 PMC6495537 30824527

[B43] Lipnick S. L. Agniel D. M. Aggarwal R. Makhortova N. R. Finlayson S. G. Brocato A. . (2019). Systemic nature of spinal muscular atrophy revealed by studying insurance claims. PLoS ONE 14:e0213680. doi: 10.1371/journal.pone.0213680 PMC6417721 30870495

[B44] Maeda K. Chong P. F. Yamashita F. Akamine S. Kawakami S. Saito K. . (2019). Global central nervous system atrophy in spinal muscular atrophy type 0. Ann. Neurol. 86, 801–802. doi: 10.1002/ana.25596 31502271

[B45] Mainster M. A. (1990). The fractal properties of retinal vessels: embryological and clinical implications. Eye 4, 235–241. doi: 10.1038/eye.1990.33 2323476

[B46] Matesanz S. E. Curry C. Gross B. Rubin A. I. Linn R. Yum S. W. . (2020). Clinical course in a patient with spinal muscular atrophy type 0 treated with nusinersen and onasemnogene abeparvovec. J. Child Neurol. 35, 717–723. doi: 10.1177/0883073820928784 32515646

[B47] Maxwell G. K. Szunyogova E. Shorrock H. K. Gillingwater T. H. Parson S. H. (2018). Developmental and degenerative cardiac defects in the Taiwanese mouse model of severe spinal muscular atrophy. J. Anat. 232, 965–978. doi: 10.1111/joa.12793 PMC5978979 29473159

[B48] Merkel S. F. Andrews A. M. Lutton E. M. Mu D. Hudry E. Hyman B. T. . (2017). Trafficking of adeno-associated virus vectors across a model of the blood–brain barrier; a comparative study of transcytosis and transduction using primary human brain endothelial cells. J. Neurochem. 140, 216–230. doi: 10.1111/jnc.13861 PMC5298820 27718541

[B49] Messina S. Sframeli M. (2020). New treatments in spinal muscular atrophy: positive results and new challenges. J. Clin. Med. 9, 1–16. doi: 10.3390/jcm9072222 PMC7408870 32668756

[B50] Møller P. Moe N. Saugstad O. D. Skullerud K. Velken M. Berg K. . (1990). Spinal muscular atrophy type I combined with atrial septal defect in three sibs. Clin. Genet. 38, 81–83. doi: 10.1111/j.1399-0004.1990.tb03553.x 2208769

[B51] Mou X. Leeman S. M. Roye Y. Miller C. Musah S. (2024). Fenestrated endothelial cells across organs: insights into kidney function and disease. Int. J. Mol. Sci. 25:9107. doi: 10.3390/ijms25169107 PMC11354928 39201792

[B52] Mukund K. Subramaniam S. (2019). Skeletal muscle: a review of molecular structure and function, in health and disease. Wiley Interdiscip. Rev. Syst. Biol. Med. 12:e1462. doi: 10.1002/wsbm.1462 PMC6916202 31407867

[B53] Müschen L. H. Osmanovic A. Binz C. Jendretzky K. F. Ranxha G. Bronzlik P. . (2021). Cerebrospinal fluid parameters in antisense oligonucleotide-treated adult 5q-spinal muscular atrophy patients. Brain Sci. 11, 1–14. doi: 10.3390/brainsci11030296 PMC7996901 33652830

[B54] Nair N. N. Kline R. A. Boyd I. Anikumar M. Thomson A. Lamont D. J. . (2025). Alterations in cardiac function correlate with a disruption in fatty acid metabolism in a mouse model of SMA. Hum. Mol. Genet. 34:547. doi: 10.1093/hmg/ddaf006 PMC11891873 39810393

[B55] Narver H. L. Kong L. Burnett B. G. Choe D. W. Bosch-Marcé M. Taye A. A. . (2008). Sustained improvement of spinal muscular atrophy mice treated with trichostatin A plus nutrition. Ann. Neurol. 64, 465–470. doi: 10.1002/ana.21449 PMC10103738 18661558

[B56] Neil E. E. Bisaccia E. K. (2019). Nusinersen: a novel antisense oligonucleotide for the treatment of spinal muscular atrophy. J. Pediatr. Pharmacol. Ther. 24, 194–203. doi: 10.5863/1551-6776-24.3.194 PMC6510522 31093018

[B57] Nery F. C. Siranosian J. J. Rosales I. Deguise M.-O. Sharma A. Muhtaseb A. W. . (2019). Impaired kidney structure and function in spinal muscular atrophy. Neurol. Genet. 5, 1–11. doi: 10.1212/NXG.0000000000000353 PMC6705648 31517062

[B58] Ottesen E. W. Singh N. N. Luo D. Kaas B. Gillette B. J. Seo J. . (2023). Diverse targets of SMN2-directed splicing-modulating small molecule therapeutics for spinal muscular atrophy. Nucleic Acids Res. 51, 5948–5980. doi: 10.1093/nar/gkad259 PMC10325915 37026480

[B59] Passini M. A. Bu J. Richards A. M. Kinnecom C. Sardi S. P. Stanek L. M. . (2011). Antisense oligonucleotides delivered to the mouse CNS ameliorate symptoms of severe spinal muscular atrophy. Sci. Transl. Med. 3:72ra18. doi: 10.1126/scitranslmed.3001777 PMC3140425 21368223

[B60] Pluta W. Lubkowska A. Dudzińska W. (2025). Vascular endothelium in health and disease: structure, function, assessment and role in metabolic disorders. Vasc. Health Risk Manag. 21, 729–747. doi: 10.2147/VHRM.S519426 PMC12415623 40927226

[B61] Poirier A. Weetall M. Heinig K. Bucheli F. Schoenlein K. Alsenz J. . (2018). Risdiplam distributes and increases SMN protein in both the central nervous system and peripheral organs. Pharmacol. Res. Perspect. 6, 1–12. doi: 10.1002/prp2.447 PMC6262736 30519476

[B62] Ramos D. M. D'Ydewalle C. Gabbeta V. Dakka A. Klein S. K. Norris D. A. . (2019). Age-dependent SMN expression in disease-relevant tissue and implications for SMA treatment. J. Clin. Invest. 129, 4817–4831. doi: 10.1172/JCI124120 PMC6819103 31589162

[B63] Ray A. Maharana K. C. Meenakshi S. Singh S. (2023). Endothelial dysfunction and its relation in different disorders: recent update. Health Sci. Rev. 7:100084. doi: 10.1016/j.hsr.2023.100084

[B64] Reilly A. Deguise M. O. Beauvais A. Yaworski R. Thebault S. Tessier D. R. . (2022). Central and peripheral delivered AAV9-SMN are both efficient but target different pathomechanisms in a mouse model of spinal muscular atrophy. Gene Ther. 29, 544–554. doi: 10.1038/s41434-022-00338-1 35462564

[B65] Riessland M. Ackermann B. Förster A. F. Jakubik M. Hauke J. Garbes L. . (2010). SAHA ameliorates the SMA phenotype in two mouse models for spinal muscular atrophy. Hum. Mol. Genet. 19, 1492–1506. doi: 10.1093/hmg/ddq023 20097677

[B66] Rudnik-Schöneborn S. Heller R. Berg C. Betzler C. Grimm T. Eggermann T. . (2008). Congenital heart disease is a feature of severe infantile spinal muscular atrophy. J. Med. Genet. 45, 635–638. doi: 10.1136/jmg.2008.057950 18662980

[B67] Rudnik-Schöneborn S. Vogelgesang S. Armbrust S. Graul-Neumann L. Fusch C. Zerres K. (2010). Digital necroses and vascular thrombosis in severe spinal muscular atrophy. Muscle Nerve 42, 144–147. doi: 10.1002/mus.21654 20583119

[B68] Salmin F. Albamonte E. Morettini V. Gagliano N. Mercuri E. Sansone V. A. (2019). Resolution of skin necrosis after nusinersen treatment in an infant with spinal muscular atrophy. Muscle Nerve 59, E42–E44. doi: 10.1002/mus.26457 30811610

[B69] Shababi M. Habibi J. Ma L. Glascock J. J. Sowers J. R. Lorson C. L. (2012). Partial restoration of cardio-vascular defects in a rescued severe model of spinal muscular atrophy. J. Mol. Cell. Cardiol. 52, 1074–1082. doi: 10.1016/j.yjmcc.2012.01.005 PMC3327806 22285962

[B70] Shababi M. Lorson C. L. Rudnik-Schöneborn S. S. (2014). Spinal muscular atrophy: a motor neuron disorder or a multi-organ disease? J. Anat. 224, 15–28. doi: 10.1111/joa.12083 PMC3867883 23876144

[B71] Signoria I. Zwartkruis M. M. Geerlofs L. Perenthaler E. Faller K. M. E. James R. . (2024). Patient-specific responses to SMN2 splice-modifying treatments in spinal muscular atrophy fibroblasts. Mol. Ther. Methods Clin. Dev. 32:101379. doi: 10.1016/j.omtm.2024.101379 PMC11626024 39655308

[B72] Silver I. Erecitiska M. (1998). Oxygen and ion concentrations in normoxic and hypoxic brain cells. Adv. Exp. Med. Biol. 454, 7–16. doi: 10.1007/978-1-4615-4863-8_2 9889871

[B73] Singh R. N. Ottesen E. W. Singh N. N. (2020). The first orally deliverable small molecule for the treatment of spinal muscular atrophy. Neurosci. Insights 15, 1–11. doi: 10.1177/2633105520973985 PMC7691903 33283185

[B74] Sintusek P. Catapano F. Angkathunkayul N. Marrosu E. Parson S. H. Morgan J. E. . (2016). Histopathological defects in intestine in severe spinal muscular atrophy mice are improved by systemic antisense oligonucleotide treatment. PLoS ONE 11, 1–15. doi: 10.1371/journal.pone.0155032 PMC4862622 27163330

[B75] Somers E. Lees R. D. Hoban K. Sleigh J. N. Zhou H. Muntoni F. . (2016). Vascular defects and spinal cord hypoxia in spinal muscular atrophy. Ann. Neurol. 79, 217–230. doi: 10.1002/ana.24549 26506088

[B76] Somers E. Stencel Z. Wishart T. M. Gillingwater T. H. Parson S. H. (2012). Density, calibre and ramification of muscle capillaries are altered in a mouse model of severe spinal muscular atrophy. Neuromuscul. Disord. 22, 435–442. doi: 10.1016/j.nmd.2011.10.021 22153987

[B77] Stahl A. Connor K. M. Sapieha P. Chen J. Dennison R. J. Krah N. M. . (2010). The mouse retina as an angiogenesis model. Invest. Ophthalmol. Vis. Sci. 51:2813. doi: 10.1167/iovs.10-5176 PMC2891451 20484600

[B78] Thomson A. K. Somers E. Powis R. A. Shorrock H. K. Murphy K. Swoboda K. J. . (2017). Survival of motor neurone protein is required for normal postnatal development of the spleen. J. Anat. 230, 337–346. doi: 10.1111/joa.12546 PMC5244459 27726134

[B79] Valori C. F. Ning K. Wyles M. Mead R. J. Grierson A. J. Shaw P. J. . (2010). Systemic delivery of scAAV9 expressing SMN prolongs survival in a model of spinal muscular atrophy. Sci. Transl. Med. 2:35ra42. doi: 10.1126/scitranslmed.3000830 20538619

[B80] Virata M. C. A. Abello R. M. R. Brillante E. M. B. Corneta M. B. Virata M. C. A. Abello R. M. R. . (2025). SMN2 as a therapeutic target in spinal muscular atrophy: advances in gene expression modulation. J. Transl. Genet. Genom. 9, 268–285. doi: 10.20517/jtgg.2025.56

[B81] Wang X. He B. (2024). Endothelial dysfunction: molecular mechanisms and clinical implications. Med. Comm. 5:e651. doi: 10.1002/mco2.651 PMC11261813 39040847

[B82] Watzlawik J. O. Kahoud R. J. O'Toole R. J. White K. A. M. Ogden A. R. Painter M. M. . (2015). Abbreviated exposure to hypoxia is sufficient to induce CNS dysmyelination, modulate spinal motor neuron composition, and impair motor development in neonatal mice. PLoS ONE 10:e0128007. doi: 10.1371/journal.pone.0128007 PMC4447462 26020269

[B83] Weissman A. S. Kennedy K. R. Powell M. R. Davis L. S. (2021). Skin necrosis in spinal muscular atrophy: case report and review of the literature. Pediatr. Dermatol. 38, 632–636. doi: 10.1111/pde.14538 33619801

[B84] Wijngaarde C. A. Blank A. C. Stam M. Wadman R. I. Van Den Berg L. H. Van Der Pol W. L. (2017). Cardiac pathology in spinal muscular atrophy: a systematic review. In Orphanet Journal of Rare Diseases, Vol. 12, No. 1 (London, UK: BioMed Central), 67. doi: 10.1186/s13023-017-0613-5 PMC5387385 28399889

[B85] Winkler E. A. Bell R. D. Zlokovic B. V. (2011). Central nervous system pericytes in health and disease. In Nature Neuroscience, Vol. 14, No. 11. (London, UK: NIH Public Access), 1398–1405. doi: 10.1038/nn.2946 PMC4020628 22030551

[B86] Wurster C. D. Koch J. C. Cordts I. Dreyhaupt J. Otto M. Uzelac Z. . (2019). Routine cerebrospinal fluid (CSF) parameters in patients with spinal muscular atrophy (SMA) treated with nusinersen. Front. Neurol. 10:1179. doi: 10.3389/fneur.2019.01179 PMC6854024 31787927

[B87] Yeo C. J. J. Darras B. T. (2020). Overturning the paradigm of spinal muscular atrophy as just a motor neuron disease. Pediatr. Neurol. 109, 12–19. doi: 10.1016/j.pediatrneurol.2020.01.003 32409122

[B88] Yeo C. J. J. Darras B. T. (2021). Yeo and Darras: extraneuronal phenotypes of spinal muscular atrophy. Ann. Neurol. 89, 24–26. doi: 10.1002/ana.25930 PMC7756714 33051879

[B89] Yu F. Pua D. K. Zaita B. Zelikovich A. S. Li J. Keselman D. J. (2026). Spinal muscular atrophy: advances in diagnosis, treatment, and emerging therapies. Curr. Treat. Options Neurol. 28:2. doi: 10.1007/s11940-026-00863-z

[B90] Yu L. Liu L. (2024). Exploration of adverse events associated with risdiplam use: retrospective cases from the US Food and Drug Administration Adverse Event Reporting System (FAERS) database. PLoS ONE 19:e0298609. doi: 10.1371/journal.pone.0298609 PMC10906863 38427665

[B91] Zeppieri M. Marsili S. Enaholo E. S. Shuaibu A. O. Uwagboe N. Salati C. . (2023). Optical coherence tomography (OCT): a brief look at the uses and technological evolution of ophthalmology. Medicina 59:2114. doi: 10.3390/medicina59122114 PMC10744394 38138217

[B92] Zhang W. Yin Y. Yang D. Liu M. Ye C. Yan R. . (2024). Comprehensive analysis of adverse events associated with onasemnogene abeparvovec (Zolgensma) in spinal muscular atrophy patients: insights from FAERS database. Front. Pharmacol. 15:1475884. doi: 10.3389/fphar.2024.1475884 PMC11747325 39840097

[B93] Zhong Z. J. Zheng P. M. Dou H. H. Wang J. G. (2023). Adverse events in the treatment of spinal muscular atrophy in children and adolescents with nusinersen: a systematic review and meta-analysis. Front. Pediatr. 11:1152318. doi: 10.3389/fped.2023.1152318 PMC10167028 37181426

[B94] Zhou H. Hong Y. Scoto M. Thomson A. Pead E. MacGillivray T. . (2022). Microvasculopathy in SMA is driven by a reversible autonomous endothelial cell defect. J. Clin. Invest. 132, 1–15. doi: 10.1172/JCI153430 PMC9621128 36099045

[B95] Zhou H. Janghra N. Mitrpant C. Dickinson R. L. Anthony K. Price L. . (2013). A novel morpholino oligomer targeting ISS-N1 improves rescue of severe spinal muscular atrophy transgenic mice. Hum. Gene Ther. 24, 331–342. doi: 10.1089/hum.2012.211 PMC3609631 23339722

[B96] Zhou H. Meng J. Marrosu E. Janghra N. Morgan J. Muntoni F. (2015a). Repeated low doses of morpholino antisense oligomer: an intermediate mouse model of spinal muscular atrophy to explore the window of therapeutic response. Hum. Mol. Genet. 24, 6265–6277. doi: 10.1093/hmg/ddv329 PMC4614699 26264577

[B97] Zhou H. Ying H. Scoto M. Brogan P. Parson S. Muntoni F. (2015b). Microvascular abnormality in spinal muscular atrophy and its response to antisense oligonucleotide therapy. Neuromuscul. Disord. 25:S193. doi: 10.1016/j.nmd.2015.06.038

